# A novel method to predict the haemoglobin concentration after kidney transplantation based on machine learning: prediction model establishment and method optimization

**DOI:** 10.1186/s12911-025-03060-1

**Published:** 2025-07-08

**Authors:** Songping He, Xiangxi Li, Fangyu Peng, Jiazhi Liao, Xia Lu, Hui Guo, Xin Tan, Yanyan Chen

**Affiliations:** 1 Digital Manufacturing Equipment National Engineering Research Center, Huazhong University of Science and Technology, Wuhan, China; 2 National NC System Engineering Research Center, Huazhong University of Science and Technology, Wuhan, China; 3 Tongji Hospital, Tongji Medical College, Huazhong University of Science and Technology, Wuhan, China; 4 Institute of Organ Transplantation, Tongji Hospital, Tongji Medical College, Huazhong University of Science and Technology; Key Laboratory of Organ Transplantation, Ministry of Education; NHC Key Laboratory of Organ Transplantation; Key Laboratory of Organ Transplantation, Chinese Academy of Medical Sciences, Wuhan, China; 5 Wuhan Intelligent Equipment Industrial Institute Co Ltd, Wuhan, China; 6 Big Data and Artificial Intelligence Office, Tongji Hospital, Tongji Medical College, Huazhong University of Science and Technology, Wuhan, China

**Keywords:** Kidney transplantation, Machine learning, Clinical prediction, Missing value imputation, Error-correcting output codes

## Abstract

**Background:**

Anaemia is a common complication after kidney transplantation, and the haemoglobin concentration is one of the main criteria for identifying anaemia. Moreover, artificial intelligence methods have developed rapidly in recent years, are widely used in the medical field and have achieved good results.

**Objective:**

To optimize the process of constructing a clinical prediction model based on machine learning and improve related technologies. A classification prediction model for the haemoglobin concentration after kidney transplantation was constructed.

**Methods:**

Real-world data from 854 kidney transplant patients in a Grade A tertiary hospital were retrospectively extracted. An imputation method combining the K-nearest neighbour algorithm and multilayer perceptron was used to fill in missing values in the dataset. Recursive feature elimination and extreme gradient boosting were used to rank and screen the importance of patient features and reduce the dimensionality of the features. Before the classification prediction model was established, the number of classification categories was determined first, and the optimal ideal cluster was approximated by the ideal cluster under each classification number and the similarity between the ideal cluster and the actual cluster. Finally, five kinds of machine learning methods, random forest, extreme gradient boosting, light gradient boosting machine, linear support vector classifier and support vector machine, were used to establish classification prediction models, and error-correcting output codes were used to optimize each model. A classification prediction model for abnormal haemoglobin concentrations after kidney transplantation was constructed, and the prediction effect was verified.

**Results:**

The imputation method combining the K-nearest neighbour algorithm and multilayer perceptron has a better effect on the imputation of missing values than do the commonly used imputation methods. Among the machine learning methods used for modelling, the prediction results of the tree model are improved to a certain degree after the error-correcting output code optimization. The final model with the best effect is optimized extreme gradient boosting, and the prediction accuracies before and after model optimization are 85.98% and 87.22%, respectively.

**Conclusions:**

The accuracy of the machine learning classification prediction model established by the optimized modelling method and process reached 87.22%, which can assist doctors in preoperative risk prediction.

**Supplementary Information:**

The online version contains supplementary material available at 10.1186/s12911-025-03060-1.

## Introduction

Kidney transplantation is the most effective way to treat end-stage renal disease [1], and statistical studies have shown that the quality of life of patients with end-stage renal disease after kidney transplantation is improved [2]. Anaemia is a common complication after kidney transplantation, with an incidence of approximately 30–40%. There are various causes of anaemia after kidney transplantation. The most common of these methods is delayed graft function (DGF) after kidney transplantation [3]. In 2009, the Global Renal Outcomes Organization (KDIGO) Guidelines for kidney transplantation defined anaemia after kidney transplantation as haemoglobin (Hb) < 135 g/L for men and < 120 g/L for women. In 2007, the World Health Organization (WHO)/American Transplantation Society (AST) defined anaemia after kidney transplantation as Hb < 130 g/L in men and < 120 g/L in women. The haemoglobin concentration of patients after kidney transplantation is the key medical test index for the diagnosis of anaemia after kidney transplantation. Therefore, the effective prediction of the HB concentration after kidney transplantation is highly important for auxiliary clinical diagnosis and risk assessment after kidney transplantation.

With the rapid development of computer and artificial intelligence technologies, related technologies in artificial intelligence have been widely used in the medical field and have played important roles in assisting in the clinical diagnosis of medical treatment. Research has shown that machine learning methods can deeply mine the value of complex and large amounts of medical data, which is difficult to achieve manually [4]. Compared with traditional decision-making methods that rely on experience, the application of machine learning can identify connections among the rich but complicated clinical features in electronic medical records, make disease predictions, provide the predicted results as important information to doctors, help doctors correctly assess surgical risk, and take intervention measures in advance. Therefore, this approach can effectively reduce postoperative risk [5]– [6].

At present, several clinical prediction models based on machine learning exist to predict the risk outcome after kidney transplantation, but the main target is whether there are serious outcomes, such as DGF, acute rejection (AR) and death after transplantation [7–12]. In addition, owing to the relatively simple modelling process, insufficient data preprocessing and feature engineering, and traditional prediction algorithms [13–17], there is still room for further improvement in the prediction effects of some current studies based on real-world data. This study further optimized the process of building a clinical prediction classification model based on machine learning and improved the existing machine learning algorithms using error-correcting output codes (ECOCs) to improve the prediction accuracy, macro average F1 score and micro average area under the curve (AUC) of the prediction model.

## Method

### Sample establishment

This study is a retrospective study based on a real-world dataset [18]. All kidney transplantation operations were performed in a Grade A tertiary hospital in Wuhan, whose Organ Transplantation Institute is the largest comprehensive medical service and research institution specializing in the clinical research of organ transplantation in China. To date, more than 6,000 cases of kidney transplantation have been carried out here. From the perspective of data integrity and availability, electronic case data from 958 kidney transplant patients in this hospital from January 2017 to June 2019 were initially included. The identification information in the patient case data was desensitized, and the patients were renumbered to obtain exemption from patient informed consent and to fully protect patient privacy. To ensure the quality of the data and the pertinence of the study, the following data screening conditions were used within the above selected scope: (1) the kidney transplant donors and recipients were all adults; (2) the kidney transplantation was a single kidney transplantation; (3) the survival time of the graft after the operation was more than 365 days; (4) the postoperative patient follow-up time was greater than 30 days; and (5) the number of feature deletions in each case was not more than 30%. Ultimately, 854 patients were included in the study.

### Missing data processing

In this study, a missing value filling method integrating the K-nearest neighbour (KNN) algorithm and multilayer perceptron (MLP) was adopted. All the features were divided into three categories according to their clinical significance and probability of missing data: “no missing”, “not easy to lose” and “relatively easy to lose”. Table 1 shows the specific classification of the three feature types. The “no missing” feature refers to the basic information of cases and preoperative medical examination information, among other criteria. In rare cases, there was a missing feature, and it was not used as the object to fill in the missing data value in this study. Features that were classified as “not easy to miss” or “relatively easy to miss” were classified based on their respective probabilities of being missing. Considering the characteristics of the filling algorithm, KNN and MLP were used to fill the missing values. The specific filling process is as follows: First, KNN is used to fill in “not easy to miss” features. At this time, the longitudinal information of the feature data table is considered, that is, the relationship between the same features in different cases. Moreover, owing to the small amount of missing data of “not easy to miss” features, the problem of low accuracy in predicting rare categories by the KNN algorithm can be better avoided when the reference data are small and the sample is unbalanced. Then, MLP is used to fill in the missing values of the remaining “relatively easy to miss” features. This method considers the horizontal information of the feature data table, that is, the relationship between different features of the same case. Based on the feature variables used in KNN filling, the features that have been filled by the KNN algorithm are added as the feature variables to predict and fill in the remaining “relatively easy to miss” feature data. When MLP is used for iterative filling, the feature completed in the previous filling becomes the feature variable of this filling; that is, the data column of the nth feature filled by the MLP algorithm for the nth time is incorporated into a new feature variable to predict and fill the *n* + 1 feature when the *n* + 1 feature is filled.

The fusion method also considers the relationship between the same features of different cases in the feature data table, that is, the vertical relationship information of the data table and the relationship between different features of a case, that is, the horizontal relationship information of the data table. At the same time, the features were classified according to the clinical significance and missing probability of each feature and were filled with different algorithms. The characteristics of the features and the characteristics of the algorithms were fully considered to ensure that the features and algorithms fit each other. Moreover, the longitudinal association information and horizontal association information of the data table were considered simultaneously.

The advantage of this method is that by integrating the KNN and MLP methods, information between the same features of different samples in the feature table (vertical information of the feature table) and information between different features of the same sample (horizontal information of the feature table) can be used simultaneously. KNN is chosen because it fully considers the vertical information of the feature list in principle. The selection of MLP is relatively flexible; its main purpose is to consider the information between different features of the same sample (horizontal information of the feature table), and most of the prediction methods, such as logistic regression and support vector machine, can actually achieve this purpose. Therefore, the greatest value of this fusion method is not to realize the combination of the KNN and MLP algorithms but to provide a way to combine the vertical and horizontal information of the feature list using the missing attributes of medical characteristics to realize the use of commonly employed prediction algorithms in the task of missing value filling. The effect of fusion is also compared with that of KNN only for missing value filling, and the results demonstrate that it is feasible to introduce the prediction algorithm into the missing value filling task based on the missing value attributes of medical characteristics.

**Table 1:** Details of feature type classification

Feature type	Missing probability	Features	Method
No missing	Hardly	Sex, age, height, weight, BMI, weight change, disease history, etc.	None
Not easy to miss	Low	Mean and extreme values of each index of blood routine examination before operation and within 30 days after surgery, medication situation, etc.	KNN
Relatively easy to miss	Relatively high	Time of reaching the maximum value of a blood routine examination, result of a urine test in the last week, etc.	MLP

In the verification phase of the filling effect of the method, the no-missing dataset is first composed of cases with no missing data. The nonmissing dataset is then subjected to 10% random deletion to simulate the worst case of missing data. That is, when the data are randomly deleted, all the feature data of a certain case except the basic information and preoperative medical examination information that rarely have missing features are deleted. In the case of the most serious missing data, the random missing dataset was used to test the filling effect of three methods: mean filling, KNN filling and KNN–MLP joint filling. The real data of cases with missing data and the mean Euclidean distance of filling data in N-dimensional space were selected as the evaluation criterion, where n is the number of features to be filled. The smaller the Euclidean distance is, the better the effect of the corresponding method; that is, the filled data are closer to the real data. The test results are shown in Table 2 and indicate that the two machine learning methods have significantly better effects than traditional mean filling does. Therefore, the KNN–MLP fusion filling method used in this study further improved the effect of KNN filling compared with the commonly used KNN filling method in machine learning.

**Table 2:** Comparison of the effects of different missing value filling methods

Methods	Considering the differences between different cases	Considering the connection between different characteristics of a case	Filling effect (Euclidean distance)
Mean padding	NO	NO	3.902
KNN	YES	NO	2.944
KNN–MLP Method	YES	YES	2.905

### Sample equalization

In classification prediction, the imbalance of sample labels of datasets leads to deviations in classification results, reducing the classification prediction accuracy of the classification model. To solve this problem, adaptive comprehensive oversampling (ADASYN) is used to perform data balancing processing on the datasets.

The prediction labels were preclassified into three categories: (1) the haemoglobin concentration 30 days after renal transplantation was within the normal range, that is, the haemoglobin concentration was within the range of 115–150 g/L, and the label was set to 0; (2) the haemoglobin concentration at 30 days after renal transplantation was lower than the normal level, that is, the haemoglobin concentration was less than 115 g/L, and the label was set to 1; and (3) the haemoglobin concentration was higher than normal 30 days after kidney transplantation, that is, the haemoglobin concentration greater than 150 g/L, was set to 2.

After labelling was set, the sample distribution of 854 cases was normal: low: high = 390:447:17, the sample distribution after adaptive comprehensive oversampling was normal: low: high = 390:447:454, the total data were expanded from 854 cases to 1291 cases, and the sample balance of the data was also good.

### Feature screening and importance analysis

**Fig. 1:**
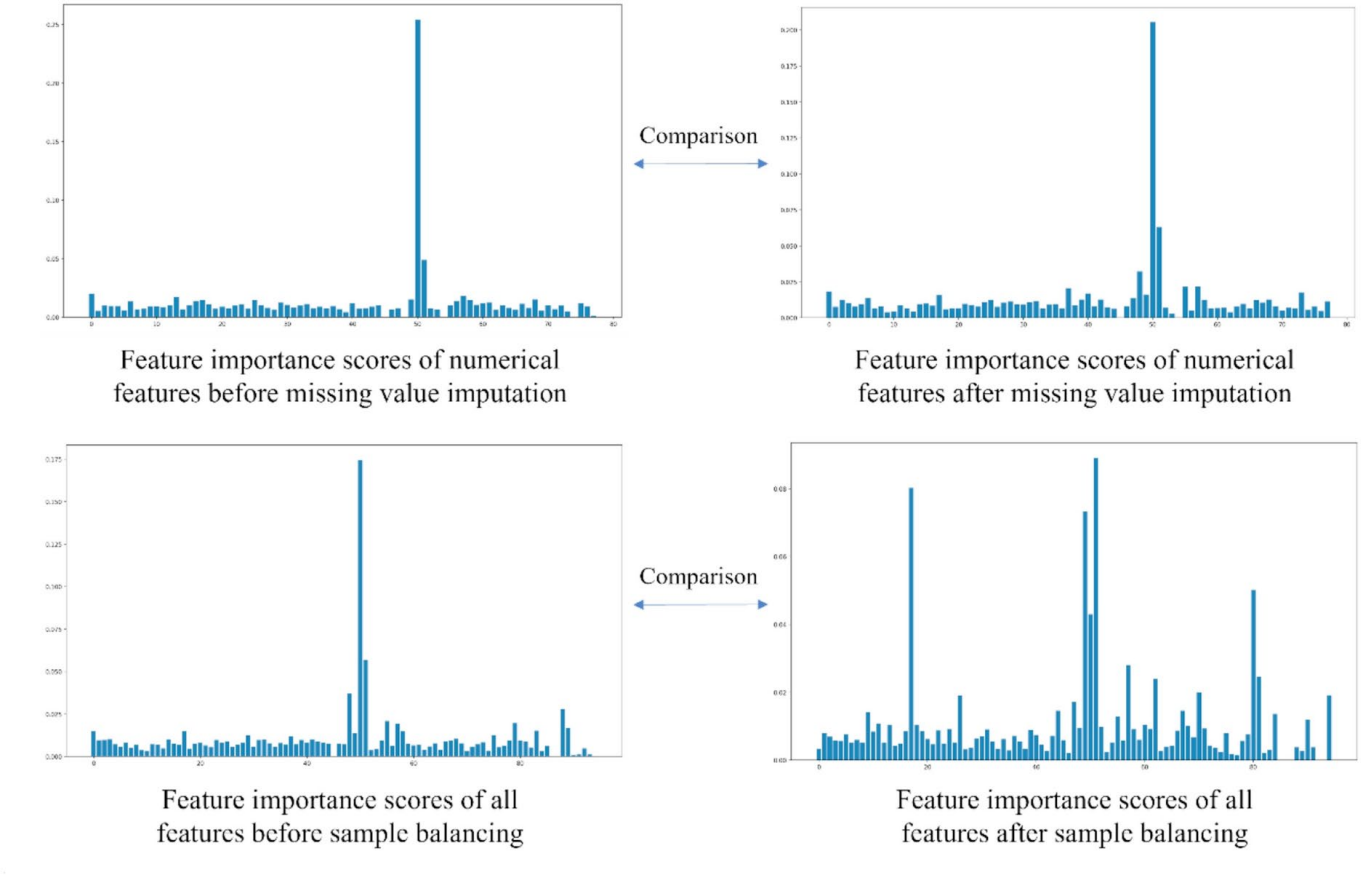
Comparison diagram of feature importance before and after data processing

In the feature screening stage, 95 features were initially included. Extreme gradient boosting (XGBoost) was used to carry out feature importance analysis for each feature, and the importance score and ranking of each feature are obtained. In this step, the data before and after the filling of missing data values and the data before and after sample balancing were sorted. The effectiveness and necessity of filling in missing data values and the processing of sample balancing were verified by observing whether there was a significant change in the importance score of each feature. The comparison results are shown in Fig. 1, which reveals that there was a significant difference in the importance of features before and after missing value filling and sample balancing, verifying the effectiveness and necessity of missing value filling and sample balancing.

**Fig. 2:**
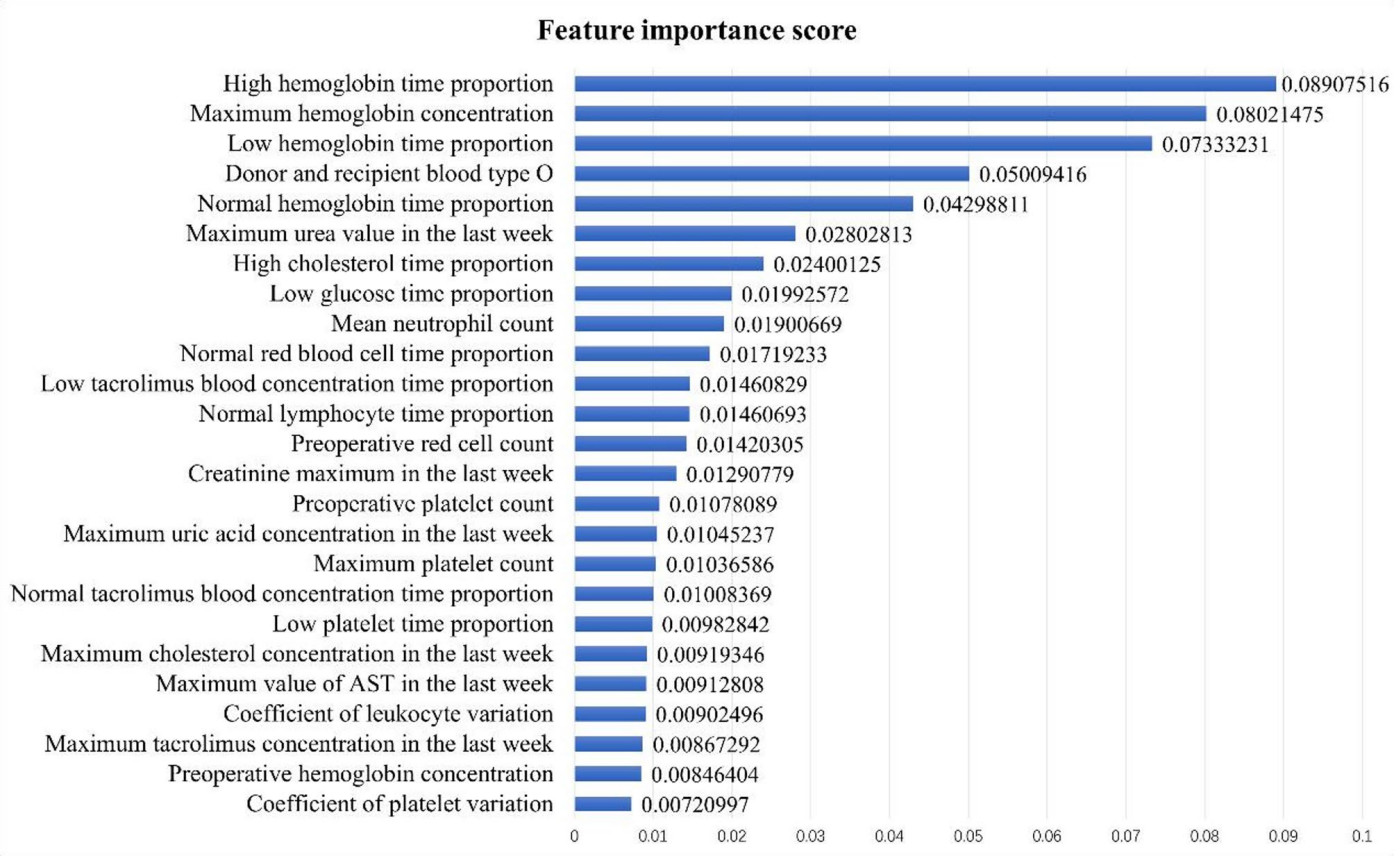
Feature screening retained features and their importance scores

After that, XGBoost was used to screen and reduce the dimension of the features based on classification accuracy. While retaining more information, as few features as possible were retained. When all 95 features were included in the model, the preliminary classification accuracy was 83.78%. Using the XGBoost algorithm alone, the best feature retention number was 22, the preliminary classification accuracy was 82.24%, and the relative accuracy loss remained within 2% of the original classification accuracy. Recursive feature elimination (RFE) was added to optimize XGBoost for feature screening and dimensionality reduction. The final result was that the top 25 features were retained. The preliminary classification accuracy reached 83.73%, and the relative accuracy loss remained within 0.1% of the original classification accuracy. Most of the information contained in the screened features was redundant information, and the initial classification accuracy decreased only slightly after the screening of these features. The 25 retained features and their respective importance values are shown in Fig. 2.

After XGBoost optimized by RFE was used to screen and retain features, LASSO regression, a commonly used feature screening method in medical statistics, was used to qualitatively verify the above feature retention results. Appendix Fig. 1 shows the correlation of features in LASSO regression as the coefficient of the penalty term changes. Appendix Fig. 2 shows the binary AUC of LASSO regression with different penalty term coefficients. Because LASSO regression is generally only used in binary classification problems, the results of this method are only used as the basis for qualitative verification of the feature screening results. The verification results are shown in Table 3. As shown in Tables 3 and 21 of the 25 screened features appeared at least once in the screening results of the other two feature screening methods, and 10 of them appeared in the screening results of the other two methods. The 25 screened features have a certain universality. To improve the generalizability of the classification prediction model, overfitting should be avoided as much as possible. The above 25 features were extracted from the initial 95 features to be incorporated into the model to avoid overfitting and reduce the computing and storage space.

**Table 3:** Mutual verification of the results of the three feature screening methods

RFE optimized XGBoost feature ordering (83.73%, *n* = 25)	Number of occurrences of other methods	XGBoost feature ordering (82.24%, *n* = 22)	LASSO regression feature ordering (*n* = 30)
High haemoglobin time proportion	2	High haemoglobin time proportion	Normal haemoglobin time proportion
Maximum haemoglobin concentration	2	Maximum haemoglobin concentration	Maximum urea value in the last week
Low haemoglobin time proportion	1	Low haemoglobin time proportion	Maximum creatinine in the last week
Donor and recipient blood type O	1	Donor and recipient blood type O	Coefficient of erythrocyte variation
Normal haemoglobin time proportion	2	Normal haemoglobin time proportion	Maximum haemoglobin concentration
Maximum urea value in the last week	2	Maximum urea value in the last week	Maximum EGFR in the last week
High cholesterol time proportion	2	Donor and recipient blood type AB	High haemoglobin time proportion
Low glucose time proportion	1	High cholesterol time proportion	Preoperative platelet count
Mean neutrophil count	1	Low glucose time proportion	Sex
Normal red blood cell time proportion	2	History of viral pneumonia	Coefficient of leukocyte variation
Low tacrolimus blood concentration time proportion	1	Mean neutrophil count	Low lymphocyte time proportion
Normal lymphocyte time proportion	2	Normal red blood cell time proportion	Maximum AST value in the last week
Preoperative red cell count	1	Normal lymphocyte time proportion	Time for lymphocytes to reach their minimum value
Creatinine maximum in the last week	2	Low tacrolimus blood concentration time proportion	Normal tacrolimus blood concentration time proportion
Preoperative platelet count	2	Preoperative red cell count	Minimum lymphocyte count
Maximum uric acid concentration in the last week	2	History of hypertension	Vaccination status
Maximum platelet count	1	Creatinine maximum in the last week	Normal red blood cell time proportion
Normal tacrolimus blood concentration time proportion	1	History of smoking	Time when neutrophils reach their maximum value
Low platelet time proportion	0	Preoperative platelet count	Lymphocyte count decreased
Maximum cholesterol concentration in the last week	0	Maximum uric acid concentration in the last week	High cholesterol time proportion
Maximum value of AST in the last week	1	Maximum neutrophil count	High glucose time proportion
Coefficient of leukocyte variation	1	Maximum platelet count	Coefficient of platelet variation
Maximum tacrolimus concentration in the last week	0		Weight
Preoperative haemoglobin concentration	0		History of hypertension
Coefficient of platelet variation	1		Age
			Normal lymphocyte time proportion
			Normal leukocyte time proportion
			Maximum uric acid concentration in the last week
			Low leukocyte time proportion
			Coefficient of lymphocyte variation

### Determination of the number of taxonomic categories

In the establishment of several previous clinical classification prediction models based on machine learning, the classification problem is often regarded as a binary classification problem by default; that is, the label is set to two groups, normal and abnormal, by default. The normal haemoglobin concentration reference range of the human body has both upper and lower limits. Therefore, in the establishment of the classification prediction model, this study added the link of determining the number of classification categories to the establishment process of the classic clinical prediction model and proposed a method to determine the number of classification categories.

**Fig. 3:**
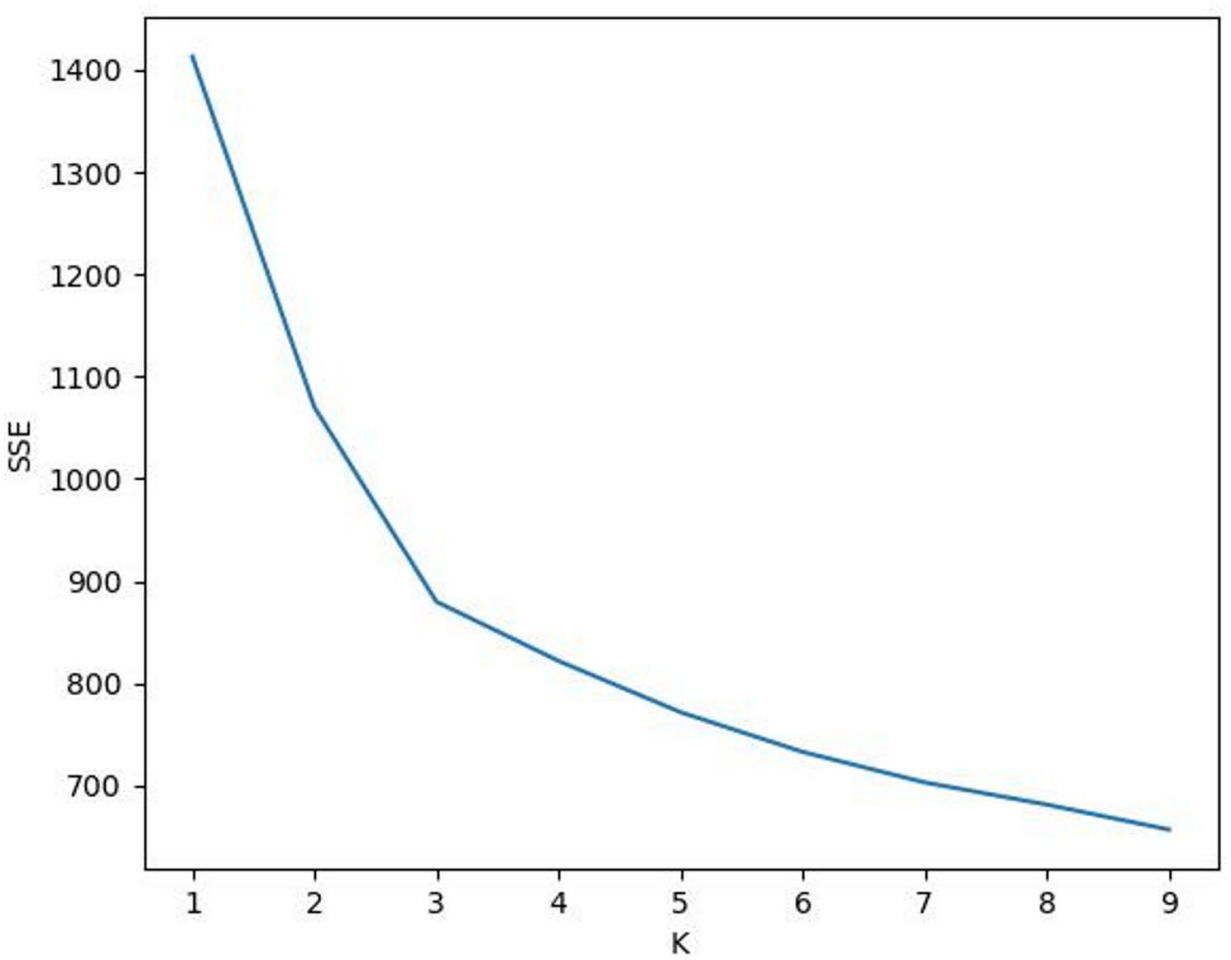
SSE curve with the number of clusters

**Fig. 4:**
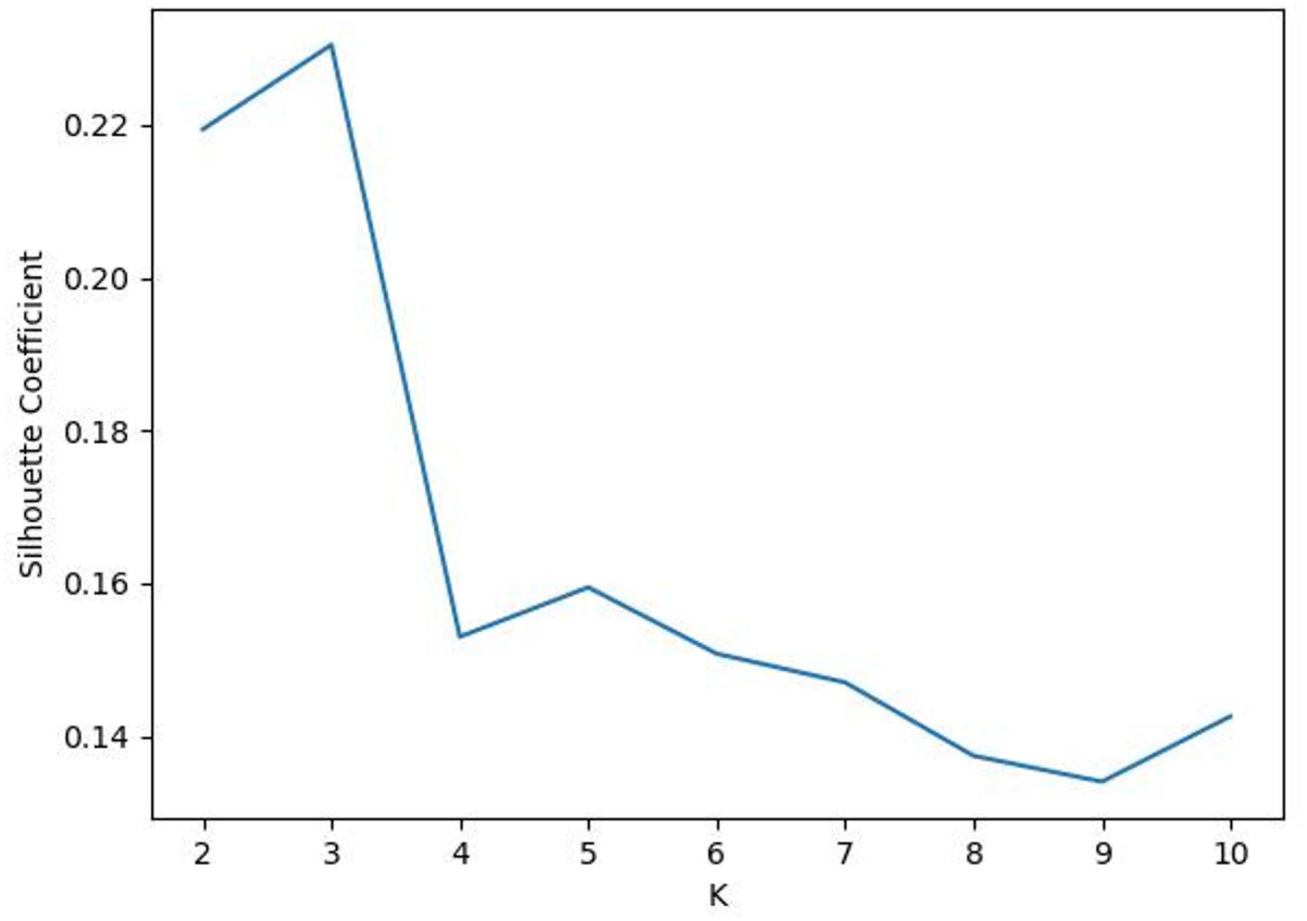
Curve of the contour coefficient changing with the number of clusters

First, the K-means clustering algorithm was used to cluster the samples without considering the clinical significance of the prediction target to obtain the ideal cluster of samples. The elbow method and contour coefficient were used to comprehensively select the best number of classification categories in the ideal cluster. Figure 3 shows the curve diagram of the sum of squared errors (SSE) changing with the number of clusters. The most obvious turning point of the curve clearly occurs when K = 3; that is, K = 3 is the best cluster determined by the elbow method. Figure 4 shows the curve of the contour coefficient changing with the number of clusters. When the contour coefficient reaches the maximum value, the number of clusters is K = 3; that is, K = 3 is the number of clusters with the highest profile coefficient. Through the above two methods, it can be concluded that without considering the clinical significance of prediction, the optimal number of clusters for ideal clustering of samples is 3.

Considering that the medical indicators targeted by clinical prediction have known limited thresholds in the clinical sense, the boundary of ideal clustering may not be the same as the threshold of clinical indicators; therefore, data labels, that is, real classification in a practical sense, will be used to divide cases and obtain actual clustering information. After that, the optimal cluster is selected comprehensively by comparing the clustering similarity between the ideal cluster and the actual cluster under each cluster number.

The specific process is as follows: According to the clinical significance of the data, two classification label settings, three classification label settings, and four classification label settings, are used to obtain the actual cluster, and the mutual information score, V-measure and other indicators are used to compare the similarity of the cluster distribution between the ideal cluster and the actual cluster under the three classification category numbers. Table 4 shows the measurement indices of the similarity of each cluster distribution of the actual and ideal clusters under the two-, three- and four-classification methods. As shown in Table 4, when the number of classification categories is 3, the similarity of the cluster distributions between the ideal and actual clusters is the highest. When the data are ideally clustered without considering the actual clinical significance, the clustering effects of the three categories are the best. Then, the actual clustering is carried out according to the clinical significance, among which the actual clustering of the three categories has the highest similarity to its ideal clustering distribution. Therefore, when the dataset is actually classified, the optimal number of classification categories should be 3. The method of determining the number of categories in this classification is named the “best approximation method”.

**Table 4:** Similarity of distribution between actual clustering and ideal clustering for each classification number

Evaluation index	Binary classification	Triple classification	Four kinds of classification
Normalized mutual information score	0.1867	0.1914	0.1835
Homogeneity score	0.1822	0.1884	0.1830
Integrity score	0.1915	0.1946	0.1840
Homogeneity and integrity harmonic mean	0.1867	0.1914	0.1835

**Fig. 5:**
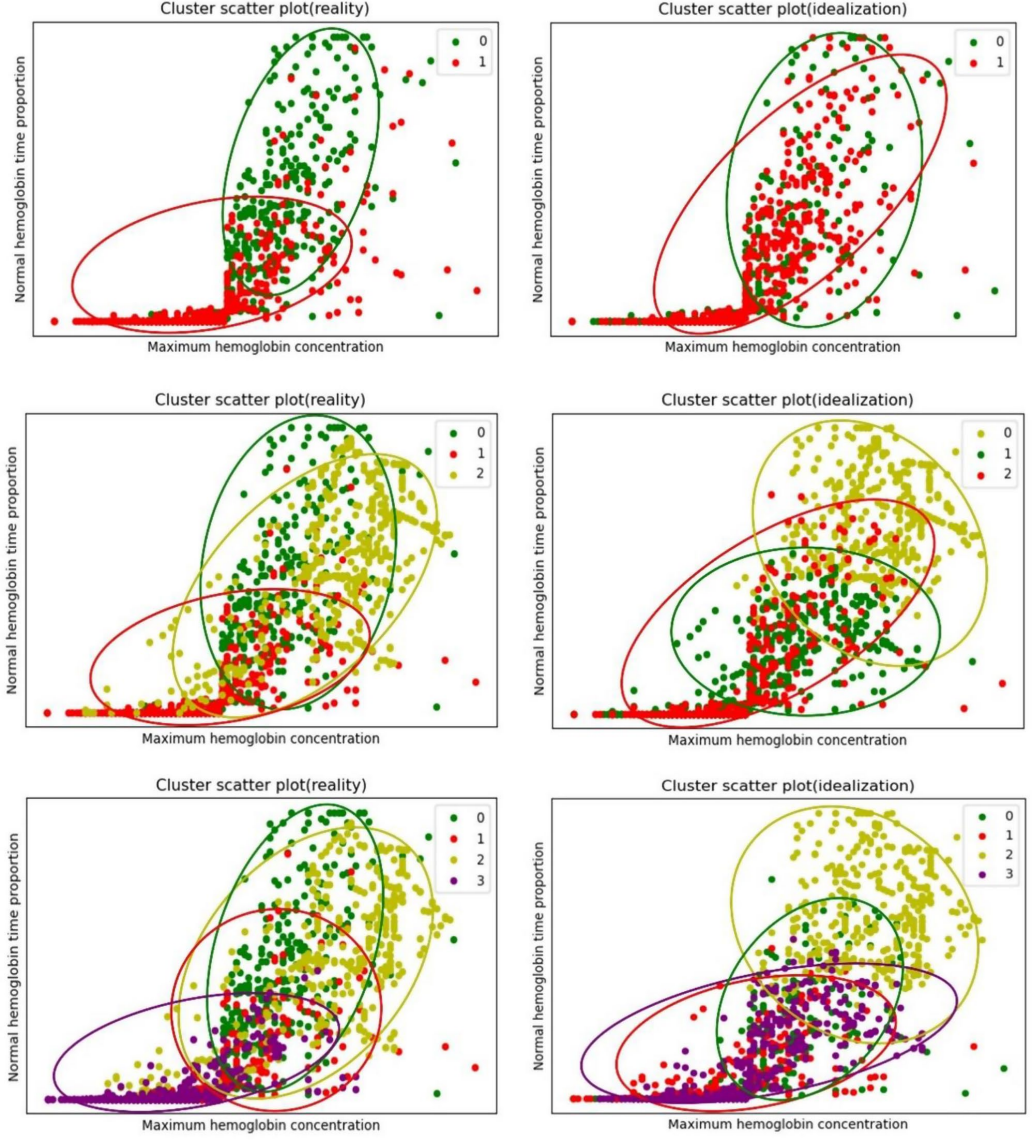
Comparison of actual clustering and ideal clustering under two-, three- and four-class classification

In Fig. 5, two features of high importance, maximum haemoglobin concentration and normal haemoglobin time proportion, are selected as the horizontal and vertical axes, respectively, to visualize the comparison between ideal clustering and actual clustering in the cases of two-, three- and four-class classification.

### Model Building and its effects

After the above procedures, a prediction model for abnormal haemoglobin concentration 30 days after kidney transplantation was established. Five machine learning algorithms, namely, random forest, XGBoost, light gradient boosting machine (LightGBM), linear support vector classification (LinearSVC) and polynomial support vector machine (Polynomial SVM), were used to construct a multiclassification prediction model for the target. Among them, the original LinearSVC and Polynomial SVM can only perform binary classification prediction; therefore, it is necessary to add ECOC to optimize these two models to perform multiclassification prediction tasks. The random forest, XGBoost and LightGBM tree models can complete multiple classification tasks by themselves. When no ECOC is added, the three tree models are used to establish classification prediction models. After that, ECOC was added to these three tree models to observe whether the classification prediction effect of the three models improved after their addition.

Error-correcting output codes (ECOC) [19] is an algorithm mainly used to solve multiclassification problems by decompressing multiclassification problems into a series of binary classification problems [20], dividing one model into multiple models. The spatial distance method is subsequently used to combine the results of each binary classification model to obtain the final result. In this study, the optimization mechanism was transferred before the multiclassification tree model to verify its effect. ECOC preprocesses the multiclass label into binary code (an array of 0s and 1s); thus, each classification label is represented by a point in the feature space. Assuming that M-bit binary code is used to represent class labels, the multiclassification problem is decomposed into M binary classification problems. First, M binary classification models are trained. When training a binary classification model, the sample of the training set is divided into a positive class and a negative class according to its binary code class (0 and 1) on the bit. Then, M models are used to predict the samples to be predicted, and the prediction result of each binary classification model will yield a 1-bit binary code (0 or 1) and finally obtain the M-bit binary code of the predicted sample. The spatial distance between the binary code of the predicted sample and the binary code of each classification label is subsequently calculated in the M-dimensional space. The classification label with the smallest spatial distance from the binary code of the predicted sample is taken as the final classification result of the predicted sample.

In terms of the algorithm mechanism, ECOC divides a model into multiple submodels, similar to the random forest model, which is composed of multiple decision tree models, which helps the model better cope with extreme data situations such as abnormal data and overfitting and improves the robustness and prediction accuracy of the model. Moreover, the way ECOC divides a multiclassification problem into several binary classification problems can reduce the computational time and space of the model compared with the way that random forests aggregate multiple models together. Compared with methods in which each decision tree is relatively independent and the final result is voted by each decision tree in the random forest, ECOC compiles the results of each submodel into a binary code and expresses them in the form of points in the feature space and then obtains the prediction results according to the distance between this point and various points in the feature space. This approach allows the results of each submodel to blend better than simply adding them together.

All the data are divided into training sets and test sets at a ratio of 8:2. Then, when training the model with the training set, tenfold stratified cross-validation is introduced to avoid overfitting during training and false high and false low results caused by individual abnormal data. In addition, the classification accuracy, macro average F1 score and micro average AUC are used to display the results. Among them, classification accuracy, as the most intuitive index, can directly reflect the classification effect of the model. The macro average F1 score and micro average AUC have relatively comprehensive evaluation ability, which can better adapt to the sample set of unbalanced categories, avoid the false high and low effects of indices caused by sample imbalance, and better reflect the comprehensive prediction ability of the model.

Table 5 shows the classification accuracy, macro average F1 score, and micro average AUC on the test set after the adjustment of various algorithms in the classification model building stage. The classification prediction effect of the tree-based model is clearly better than that of the linear model. The error correction output code also has a good optimization effect on the tree-based model. For the test set, the classification prediction accuracies of the random forest, XGBoost and LightGBM methods improved from 85.52%, 85.98% and 85.91–86.49%, 87.22% and 86.64%, respectively, after optimized error correction output coding. The macro average F1 scores increased from 85.00%, 85.45% and 85.41–85.87%, 86.70% and 86.02%, respectively. The micro average AUC increased from 89.14%, 89.49% and 89.43–89.86%, 90.42% and 89.98%, respectively. The above results indicate that the classification accuracy, macro average F1 score and micro average AUC of the three tree-based models optimized by ECOC improve correspondingly, which verifies the optimization effect of the ECOC on the tree-based model. XGBoost optimized by error-correcting output coding was selected to build the final prediction model with the best classification prediction effect. The classification accuracy was 87.22%, the macro average F1 score was 86.70%, and the micro average AUC was 90.42%.

**Table 5:** Comparison of the classification effects of each model

Algorithm	Classification accuracy (%)			Macro average F1 score (*10^− 2^)			Micro average AUC (*10^− 2^)		
	A	B	B-A	A	B	B-A	A	B	B-A
Random Forest	85.52	86.49	0.97	85.00	85.87	0.87	89.14	89.86	0.72
XGBoost	85.98	**87.22**	**1.24**	85.45	**86.70**	**1.25**	89.49	**90.42**	**0.93**
LightGBM	85.91	86.64	0.73	85.41	86.02	0.61	89.43	89.98	0.55
Linear SVC		82.82			81.71			87.11	
Polynomial SVM		77.68			76.97			83.26	

STATEMENT

A: Value of this indicator when no optimization is performed

B: Value of this indicator after ECOC optimization

B-A: Increase in this index after ECOC optimization

### Model evaluation

**Fig. 6:**
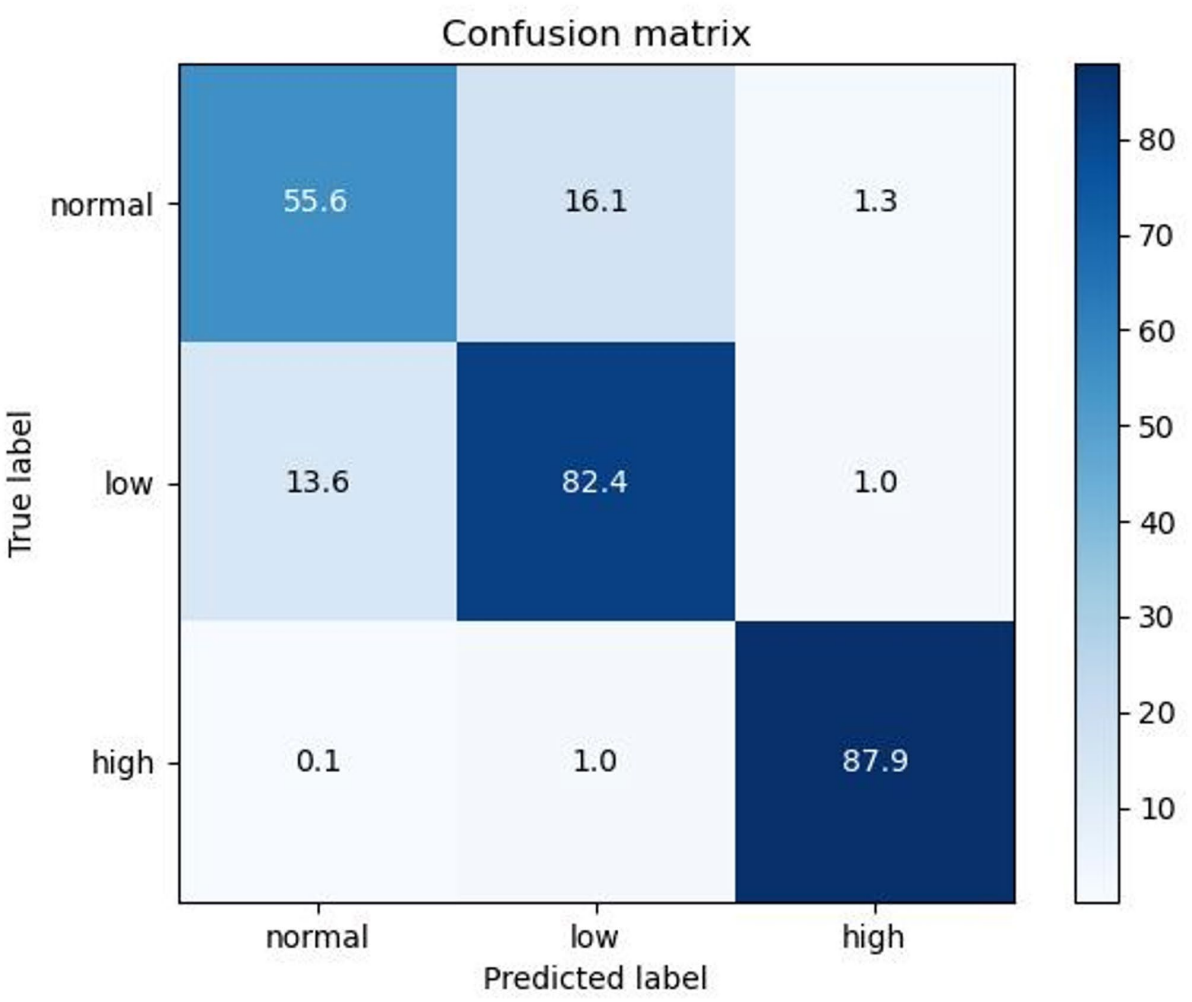
Test set confusion matrix of the classification prediction model. STATEMENT: The decimal number in the figure is because using tenfold stratified cross-validation will eventually build ten models, and the confusion matrix is plotted using their mean

XGBoost optimized by ECOC with the best prediction effect is selected as the final prediction model, and its detailed evaluation indicators in the test set are shown in Table 6. The confusion matrix is used to more concretely reflect the classification prediction effect of the model. Figure 6 shows the confusion matrix of the classification prediction model on the test set. Table 7 shows the evaluation indicators of each label on the test set, including precision, recall and F1 score, to facilitate comprehensive evaluation of the model effect based on actual clinical significance and needs.

**Table 6:** Detailed indicators of the final classification prediction model

Algorithm	Accuracy (%)	Precision (%)	Recall (%)	Macro average F1 score (*10^− 2^)	Micro average AUC (*10^− 2^)
ECOC-XGBoost	87.22	87.00	86.60	86.70	90.42

The purpose of this study was mainly to predict the risk of adverse outcomes after kidney transplantation surgery to assist doctors in quickly identifying and focusing on patients’ conditions and to predict and evaluate surgical risks in advance. Therefore, for the classification effect index of the model, in addition to the overall classification prediction accuracy, macro average F1 score and micro average AUC, the recall rates of abnormal groups should also be considered. Table 7 shows that although the overall classification accuracy reaches 87.2%, the recall rates of the low group (label 1) and the high group (label 2) are also as high as 85.3% and 98.9%, respectively, with a good effect and in line with the clinical needs targeted by the model.

**Table 7:** Evaluation indicators of each label on the test set

	Precision	Recall	F1 score	Test set number of samples
Normal Label: 0	0.803	0.762	0.781	73
Low Label: 1	0.829	0.853	0.839	97
High Label: 2	0.977	0.989	0.981	89
**Accuracy**			0.872	259
**Macro average**	0.870	0.866	0.867	259
**Weighted average**	0.872	0.871	0.872	259

## Results

### Missing value filling

In this study, a fusion filling method is proposed for the missing value problem of datasets, which combines the KNN and MLP algorithms and fully considers the missing attributes of each medical feature. Different algorithms are used to fill in the missing values of the features of different missing attributes and fully consider the characteristics of the features and the characteristics of the algorithm so that the features and algorithms can be adapted to each other. At the same time, the fusion of the KNN algorithm and MLP also considers the relationship between the same features of different cases in the feature data table and the relationship between different features of the same case and realizes the simultaneous consideration of vertical and horizontal association information in the data table. When a 10% random deletional dataset is used to test and compare the methods, the mean Euclidean distance between the filling value and the true value in the feature space of the KNN–MLP method is 2.905, which has obvious advantages over the Euclidean distance of 3.902 obtained by means of traditional statistics. Compared with the Euclidean distance of 2.944 of the KNN algorithm, which is often used to fill missing values in machine learning, there is also a certain improvement. The fusion filling algorithm has a good filling effect on the missing values of real medical data.

### Feature importance ranking

Before establishing the model, this study screened the included features to achieve dimensionality reduction. In this process, XGBoost optimized by REF was used to sort the features according to importance. The characteristics that have the greatest influence on the abnormal haemoglobin concentration 30 days after renal transplantation are the proportion of high haemoglobin time, maximum haemoglobin value, proportion of low haemoglobin time, donor and recipient type O blood, proportion of normal haemoglobin time, maximum urea value in the last week, proportion of high cholesterol time and proportion of low glucose time, among others. These results can assist doctors in clinical judgement, pay greater attention to features that are highly correlated with the haemoglobin concentration, and carry out a risk assessment of patients in the prognosis process by paying attention to these features. Moreover, these results can be used as a practical basis for studying the internal correlation mechanism between high correlation characteristics and haemoglobin concentrations.

### Model prediction results

In this study, five machine learning algorithms, random forest, XGBoost, LightGBM, LinearSVC and Polynomial SVM, were used to establish a prediction model of abnormal haemoglobin concentrations after kidney transplantation. Related optimization methods were added to establish a multiclassification prediction model for the target. The prediction results are shown in Table 5 and indicate that the prediction effects of the random forest, XGBoost and LightGBM tree model algorithms are significantly better than those of the other two methods. Moreover, when the ECOC was applied to the optimization of the random forest, XGBoost and LightGBM tree models, the classification prediction accuracies of the three algorithms increased from 85.52%, 85.98% and 85.91–86.49%, 87.22% and 86.64%, respectively. The macro average F1 scores increased from 85.00%, 85.45% and 85.41–85.87%, 86.70% and 86.02%, respectively. The micro average AUCs increased from 89.14%, 89.49% and 89.43–89.86%, 90.42% and 89.98%, respectively. These results show that the optimization significantly improves the prediction accuracy of the tree models. The best classification prediction model is the XGBoost prediction model optimized by the ECOC: its classification accuracy reaches 87.22%, the macro average F1 score reaches 86.70%, and the micro average AUC reaches 90.42%.

## Discussion

**Fig. 7:**
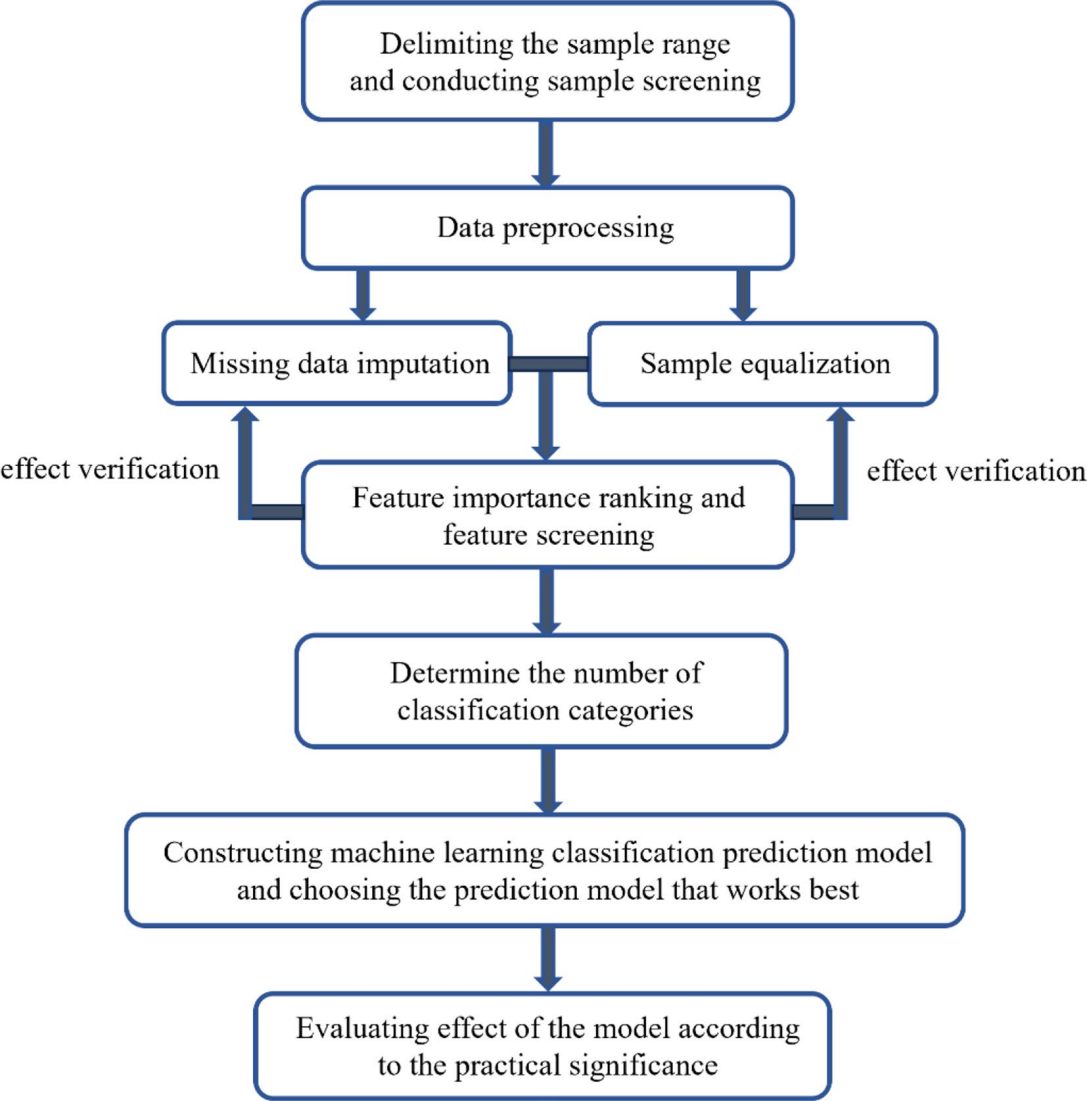
Flowchart of the model establishment

This study established a prediction model for abnormal haemoglobin concentrations 30 days after kidney transplantation based on machine learning and added relevant steps to further refine and improve the process of model establishment. Figure 7 shows the flow chart of model establishment. Moreover, in terms of missing data value filling, a fusion filling method was proposed in combination with medical characteristics. The algorithm was improved by transferring and using an error correction output code, and the prediction accuracy of the model reached 87.22% on real-world data. The recall rates of patients with low and high haemoglobin abnormalities reached 85.3% and 98.9%, respectively, and the effect was good. This model can assist doctors in making clinical judgments and predicting the risk of renal transplantation in advance.

In recent years, with the continuous development of artificial intelligence technology, increasingly complex deep learning networks have been built and used by scholars; these complex deep learning networks are commonly used in image, language, audio and other fields. However, for the prediction of tabular-based data such as real-world data in medical clinical practice, the performance of deep learning networks is relatively general. This is because tabular data have uneven characteristics, small sample sizes, large extreme values, and other delineating factors; therefore, it is difficult to find the corresponding invariants. A 2022 study showed that for tabular data, when tree-based methods such as random forest and XGBoost are used, LightGBM more easily achieves good predictions than deep learning does, and tree-based models still outperform deep learning methods for tabular data [21].

In this study, the importance ranking of the features that had the greatest impact on the abnormal haemoglobin concentration at 30 days after kidney transplantation was obtained in the feature screening stage. Some of these features were significantly correlated with the haemoglobin concentration at 30 days after kidney transplantation, such as the proportion of time with high haemoglobin and the maximum value of haemoglobin, but some features were not significantly correlated with the abnormal haemoglobin concentration, such as O blood type of the donor or recipient, the maximum urea value in the previous week, and the proportion of high cholesterol. These features, which have no obvious correlation, will provide doctors with more tips in clinical judgement. These results can also serve as a practical basis for doctors to study the internal mechanism of the connection between these features with no obvious correlation and the target.

In this study, the ECOC was transferred to the application tree model, and the prediction accuracy of the model was better improved, whereas the ECOC was mainly applied to solving multiclassification tasks using a binary classification algorithm. The principle is to decompose multiclassification problems into a series of binary classification problems, and the tree model itself can complete multiclassification tasks. The more detailed internal mechanism by which ECOC can still improve the prediction effect of the model even when it is used in the tree model still needs to be further studied.

At present, studies on machine learning-based methods for predicting the prognosis of patients after kidney transplantation have focused mainly on the prediction of severe disease, such as acute rejection (AR) and delayed graft function (DGF), and have not focused on detailed indicators, such as the haemoglobin concentration. There is also a lack of public datasets on these detailed indicators. In this study, all the data are divided into a training set and a test set at an 8:2 ratio. During the training of the model, the test set data are never included, and its information is not leaked to the model at all. Finally, the model is evaluated using a new test set to ensure the generalizability of the model.

A total of 854 cases of data were included in this study. For the sample size of input data for machine learning models, it is generally accepted that the amount of data should range from several hundred to several thousand but not be less than 300. However, there are sometimes examples of using small sample sizes to build machine learning models. In 2020, Kyle R. Jackson used the data of 140 marginal deceased donor kidneys and transplant recipients to compare the prediction accuracy of the two donor kidney scoring systems [22]. There are also examples of using large sample sizes to construct a machine learning predictive model. Yunwei Zhang used data from 3,624 patients in 2023 to develop a prediction model for the survival rate of grafts after kidney transplantation [23]. In 2018, Richard D Riley mentioned a calculation method to meet the minimum sample size requirements in his study [24]. According to the calculation method of Richard D Riley, in this study, the total number of follow-up patients was 854, the follow-up time was one year, the number of abnormal patients was 464, the abnormal rate was 54.33%, and the final number of features included in the modelling was 25. The calculated EPP of this study is (854 × 1 × 54.33%)/25 = 18.56, which conforms to the rule that the EPP is at least 10. Therefore, the amount of data in this study is larger than the minimum sample size needed.

The model established in this study has good interpretability, which is reflected mainly in the steps of feature screening and importance ranking using the XGBoost algorithm with recursive feature elimination. In this study, we did not use feature extraction methods such as neural networks or convolutions but rather chose the XGBoost algorithm to perform feature screening on the original features to better conform to the characteristics of medical prediction tasks. The features obtained using feature extraction methods such as neural networks or convolutions can be understood only by computers, which has no specific significance in actual clinical practice. When the XGBoost algorithm is used for feature screening, each feature selected is clinically meaningful and has better interpretability. In addition, these clinical features with practical significance will provide doctors with more tips when making clinical judgements. Moreover, these results can also serve as a practical basis to prompt doctors to study the internal mechanism of the connection between features that are not clearly linked and the target, which also makes the model more interpretable. In principle, the XGBoost algorithm itself has strong interpretability, mainly stemming from the Shapley value tool it uses. The Shapley value assigns a contribution value to each feature, reflecting the degree to which the feature influences the final prediction. Shapley values can provide a detailed interpretation of a single prediction, or they can show the global impact of a feature by aggregating multiple predictions. In addition, the Shapley value is consistent with the feature importance ranking of the model; that is, if the contribution of the feature increases, its Shapley value increases accordingly, which is why XGBoost is well suited for use with recursive feature elimination.

Notably, all patients in this study underwent kidney transplantation for the first time, and the causes of renal failure were not recorded. The causes of kidney failure, previous methods of kidney replacement, and previous transplant history were not included in this study. The effects of these characteristics on abnormal haemoglobin concentrations in patients after renal transplantation need further study.

The different prediction abilities of the classification prediction model for two types of abnormal haemoglobin concentrations (low and high) need to be considered and further discussed. The prediction ability of the model for high haemoglobin concentration (label 2) is greater than that for low haemoglobin concentration (label 1). First, it was believed that the overfitting of high haemoglobin concentration was caused by the oversampling of samples with high haemoglobin concentrations in the sample equalization stage. Later, tenfold stratified cross-validation was added to the model training to solve the overfitting problem, and two comprehensive indices, the macro average F1 score and the micro average AUC, were added to show the model prediction results. However, there are still some differences in the prediction ability of the models for the two kinds of abnormal conditions. At the same time, this study established a model with the same method for the data without sample-balanced oversampling to make preliminary predictions. The recall rate of the model for samples with high haemoglobin concentrations (label 2) was only 25%, which was much lower than those for the other two types of samples (64% and 86%). This is because too few high-label samples lead to inadequate training of the model, which further explains the necessity of using sample equalization in this study. The superior ability of the prediction model to predict higher haemoglobin concentrations (label 2) compared with lower haemoglobin concentrations (label 1) may be because samples with higher haemoglobin concentrations are more clearly different from other samples in some of the included features. This makes the model better able to learn the features of samples with high haemoglobin concentrations more fully so that it has better prediction ability for such samples. These findings need further study.

## Electronic supplementary material

Below is the link to the electronic supplementary material.

Supplementary Material 1

Supplementary Material 2

Supplementary Material 3

## Data Availability

The datasets used and/or analysed during the current study are available from the corresponding author on reasonable request.
