# Supplementary material for: A novel method to predict the haemoglobin concentration after kidney transplantation based on machine learning: prediction model establishment and method optimization
Source: BMC Med Inform Decis Mak. 2025 Jul 8;25:255. doi: 10.1186/s12911-025-03060-1 (PMC12236034; doi:10.1186/s12911-025-03060-1)
Supplement: Supplementary file 1 — Supplementary Material 1 [file 12911_2025_3060_MOESM1_ESM.docx]

**The figure legend for the appendix figure**

Appendix Figure 1. The changes of feature correlation with penalty term coefficients in LASSO regression

Appendix Figure 2. AUC of LASSO regression for different penalty term coefficients
