# Supplementary figures and images for: A novel method to predict the haemoglobin concentration after kidney transplantation based on machine learning: prediction model establishment and method optimization
Source: BMC Med Inform Decis Mak. 2025 Jul 8;25:255. doi: 10.1186/s12911-025-03060-1 (PMC12236034; doi:10.1186/s12911-025-03060-1)

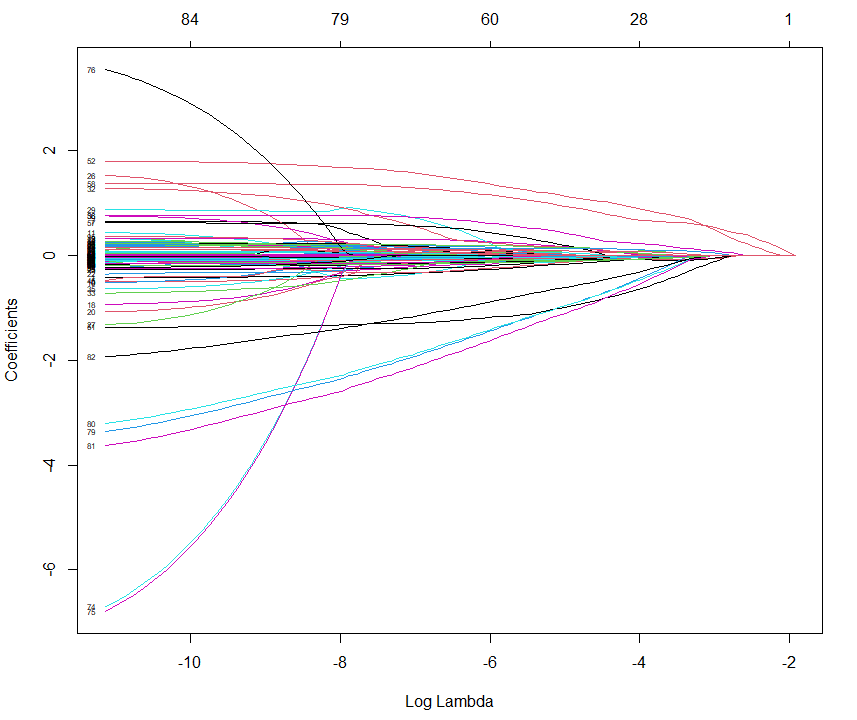

Supplement: Supplementary file 2 — Supplementary Material 2 [file 12911_2025_3060_MOESM2_ESM.png]

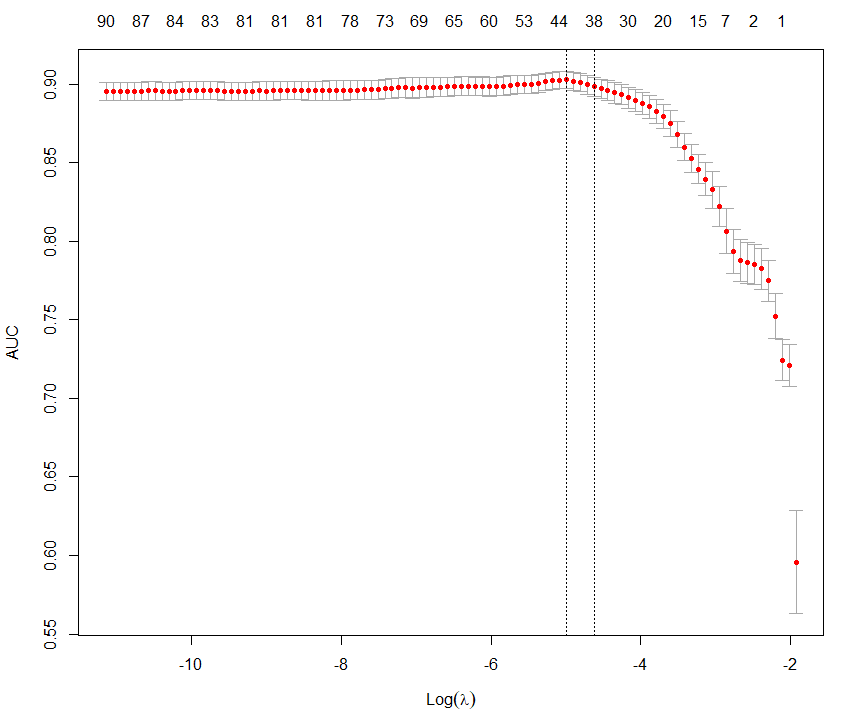

Supplement: Supplementary file 3 — Supplementary Material 3 [file 12911_2025_3060_MOESM3_ESM.png]
